# Supplementary material for: Assessing the relationship between early maladaptive schemas and interpersonal problems using interpersonal scenarios depicting rejection
Source: PLoS One. 2023 Oct 24;18(10):e0288543. doi: 10.1371/journal.pone.0288543 (PMC10597527; doi:10.1371/journal.pone.0288543)
Supplement: S1 Appendix — Friend, family and romantic vignettes developed for the study with variations based on acceptance, ambiguous and unambiguous rejection conditions. (PDF) [file pone.0288543.s001.pdf]

Appendix A: Interpersonal vignettes

Friendship scenarios

Vignette 1 - Friend

For the following scenario, please pick a close friend of yours (i.e., it can be any friend that you feel particularly close to). Imagine that the scenario refers to that friend:

Control
Imagine you are walking through your local shopping centre to buy groceries. You pass through the canned soup isle when you see one of your friends is also shopping for soup. They are facing the isle looking for something. As you begin to approach them, you smile, wave to your friend and say “hi”. As you walk past them, they wave back at you, smile, and briefly discuss their day with you. As you say goodbye, they then turn back to the isle to search for soup and you go your separate ways and continue shopping. Please take a moment to imagine this scenario as if it were happening to you right now.

Ambiguous
Imagine you are walking through your local shopping centre to buy groceries. You pass through the canned soup isle when you see one of your friends is also shopping for soup. They are facing the isle looking for something. As you begin to approach them, you smile, wave to your friend and say “hi”. As you walk past them, they don't say or do anything in response to your greeting. They still appear to be facing the isle looking for soup. Please take a moment to imagine this scenario as if it were happening to you right now.

Rejection
Imagine you are walking through your local shopping centre to buy groceries. You pass through the canned soup isle when you see one of your friends is also shopping for soup. They are facing the isle looking for something. As you begin to approach them, you smile, wave to your friend and say “hi”. As you walk past them, they look at you and roll their eyes. They then turn back to the aisle and resume looking for soup without saying anything. Please take a moment to imagine this scenario as if it were happening to you right now.

Vignette 2 – Friend

For the following scenario, please pick a close friend of yours (i.e., it can be any friend that you feel particularly close to). Imagine that the scenario refers to that friend:

Control

Imagine you are attending an old school friend's or colleague's party. You don't know most of the people there, but suddenly you see a friend you haven't seen for a while. As you begin talking to them you start to think to yourself how much you have missed seeing this friend. Excited to talk to them again, you suggest your friend should call or text you sometime soon so that you can catch up again. The next day you text your friend to suggest an idea for catching up. They text you back suggesting a date and time. Please take a moment to imagine this scenario as if it were happening to you right now.

Ambiguous

Imagine you are attending an old school friend's or colleague's party. You don't know most of the people there, but suddenly you see a friend you haven't seen for a while. As you begin talking to them you start to think to yourself how much you have missed seeing this friend. Excited to talk to them again, you suggest your friend should call or text you sometime soon so that you can catch up again. The next day you text your friend to suggest an idea for catching up. A few days later your friend still hasn't called or texted you back. Please take a moment to imagine this scenario as if it were happening to you right now.

Rejection

Imagine you are attending an old school friend's or colleague's party. You don't know most of the people there, but suddenly you see a friend you haven't seen for a while. As you begin talking to them you start to think to yourself how much you have missed seeing this friend. Excited to talk to them again, you suggest your friend should call or text you sometime soon so that you can catch up again. The next day you text your friend to suggest an idea for catching up. They text you back saying they would prefer not to catch up because they only have time for their close friends right now. Please take a moment to imagine this scenario as if it were happening to you right now.

Vignette 3 – Friend

For the following scenario, please pick a close friend of yours (i.e., it can be any friend that you feel particularly close to). Imagine that the scenario refers to that friend:

Control

Imagine it’s the weekend and you are feeling a bit bored. You are thinking about what you could do to make the most of your time. You think it might be a good idea to spend some time with a friend. You think about something you might both enjoy doing together such as going to the movies, having lunch or doing a fun activity together. You then call your friend to see if they have the time and if they are interested. Your friend answers the phone and says they’re free, saying they will see you soon. You decide to get ready and go out to meet your friend. When you go out, you both have a great time. Please take a moment to imagine this scenario as if it were happening to you right now.

Ambiguous

Imagine it’s the weekend and you are feeling a bit bored. You are thinking about what you could do to make the most of your time. You think it might be a good idea to spend some time with a friend. You think about something you might both enjoy doing together such as going to the movies, having lunch or doing a fun activity together. You then call your friend to see if they have the time and if they are interested. However, when you call nobody answers the phone. You decide to get ready and go out by yourself instead. When you go out, you see your friend laughing and spending time with another friend. Please take a moment to imagine this scenario as if it were happening to you right now.

Rejection

Imagine it’s the weekend and you are feeling a bit bored. You are thinking about what you could do to make the most of your time. You think it might be a good idea to spend some time with a friend. You think about something you might both enjoy doing together such as going to the movies, having lunch or doing a fun activity together. You then call your friend to see if they have the time and if they are interested. Your friend answers but explains that they are too busy and need to stay at home to get their work done. You decide to get ready and go out by yourself instead. When you go out, you see your friend laughing and spending time with another friend. Please take a moment to imagine this scenario as if it were happening to you right now.

Family scenarios

Vignette 4 – Family

For the following scenario, please pick a close family member of yours (i.e., it can be anyone you felt particularly close to such as your mother, father, brother, sister, grandparents or uncle/aunty). Imagine that the scenario refers to that particular family member:

Control
Imagine that you haven't seen or spoken to one of your close family members for a while. You begin to think about how much you have missed talking to them and how it's probably about time you checked in to see how they were going. You've also got lots of things you want to tell them about. You're both quite busy at the moment so you quickly call them to arrange a later time that you are both free to talk over the phone. You and your family member both agree to a time and you put that time aside in your schedule. When that time comes around you eagerly give them a call, excited to catch up. They quickly answer giving you time to catch up and have a good chat. Please take a moment to imagine this scenario as if it were happening to you right now.

Ambiguous
Imagine that you haven't seen or spoken to one of your close family members for a while. You begin to think about how much you have missed talking to them and how it's probably about time you checked in to see how they were going. You've also got lots of things you want to tell them about. You're both quite busy at the moment so you quickly call them to arrange a later time that you are both free to talk over the phone. You and your family member both agree to a time and you put that time aside in your schedule. When that time comes around you eagerly give them a call, excited to catch up. You dial their number and the phone starts ringing. The phone continues to ring out and you get transferred to their message bank. Please take a moment to imagine this scenario as if it were happening to you right now.

Rejection
Imagine that you haven't seen or spoken to one of your close family members for a while. You begin to think about how much you have missed talking to them and how it's probably about time you checked in to see how they were going. You've also got lots of things you want to tell them about. You're both quite busy at the moment so you quickly call them to arrange a later time that you are both free to talk over the phone. You and your family member both agree to a time and you put that time aside in your schedule. When that time comes around you eagerly give them a call, excited to catch up. They answer but quickly tell you they can't talk right now. Please take a moment to imagine this scenario as if it were happening to you right now.

Vignette 5 – Family

For the following scenario, please pick a close family member of yours (i.e., it can be anyone you felt particularly close to such as your mother, father, brother, sister, grandparents or uncle/aunty). Imagine that the scenario refers to that particular family member:

Control
Imagine you have been experiencing quite a lot of stress lately either at home, work or school. You are working it out and coping ok but it always makes you feel better when you can have a vent or talk to somebody else about it. One afternoon you decide to call one of your family members to update them on how you are going. When you call, your family member begins by updating you about what has been happening in their life. You listen carefully, thinking how nice it is to talk with them. When they are finished you begin updating them about how you have been going. While you are talking, you can hear your family member listening as they ask you questions about how you are coping. Please take a moment to imagine this scenario as if it were happening to you right now.

Ambiguous
Imagine you have been experiencing quite a lot of stress lately either at home, work or school. You are working it out and coping ok but it always makes you feel better when you can have a vent or talk to somebody else about it. One afternoon you decide to call one of your family members to update them on how you are going. When you call, your family member begins by updating you about what has been happening in their life. You listen carefully, thinking how nice it is to talk with them. When they are finished you begin updating them about how you have been going. While you are talking, you notice your family member hasn't said anything in a while and you can hear things moving around in the background. When they finally respond they ask you to repeat what you just said. Please take a moment to imagine this scenario as if it were happening to you right now.

Rejection
Imagine you have been experiencing quite a lot of stress lately either at home, work or school. You are working it out and coping ok but it always makes you feel better when you can have a vent or talk to somebody else about it. One afternoon you decide to call one of your family members to update them on how you are going. When you call, your family member begins by updating you about what has been happening in their life. You listen carefully, thinking how nice it is to talk with them. When they are finished you begin updating them about how you have been going. However, as you are talking you can hear them sigh and they eventually cut you off telling you "I don't want to talk about this". Please take a moment to imagine this scenario as if it were happening to you right now.

Vignette 6 – Family

For the following scenario, please pick a close family member of yours (i.e., it can be anyone you felt particularly close to such as your mother, father, brother, sister, grandparents or uncle/aunty). Imagine that the scenario refers to that particular family member:

Control
Imagine you have an upcoming special event such as birthday or a function where you are being congratulated for an achievement. In excitement for the event you invite one of your close family members to attend. You begin to feel excited and are happy to be able to share this event with the people closest to you. You are especially excited for your family member to come. When the event arrives, you begin looking around and walking through the crowds of people to see if your family member has arrived. You eventually see them in the crowd and start talking with them. Please take a moment to imagine this scenario as if it were happening to you right now.

Ambiguous
Imagine you have an upcoming special event such as birthday or a function where you are being congratulated for an achievement. In excitement for the event you invite one of your close family members to attend. You begin to feel excited and are happy to be able to share this event with the people closest to you. You are especially excited for your family member to come. When the event arrives, you begin looking around and walking through the crowds of people to see if your family member has arrived. You've been walking through the crowd for some time now and still can't see them anywhere. You text them but there is no answer. Please take a moment to imagine this scenario as if it were happening to you right now.

Rejection
Imagine you have an upcoming special event such as birthday or a function where you are being congratulated for an achievement. In excitement for the event you invite one of your close family members to attend. You begin to feel excited and are happy to be able to share this event with the people closest to you. You are especially excited for your family member to come. When the event arrives, you begin looking around and walking through the crowds of people to see if your family member has arrived. As you walk through the crowd, you receive a text from your family member telling you that they won't be able to come. Please take a moment to imagine this scenario as if it were happening to you right now.

Romantic scenarios

Vignette 7 – Romantic

For the following scenario, please imagine your partner/girlfriend or boyfriend. If you do not currently have a partner, please imagine the person from your last romantic relationship:

Control
Imagine you are heading home/going to visit your partner one afternoon. Your partner is usually home at this time. You've really been looking forward to seeing them and telling them about your day. When you finally arrive, you walk up to the front door and open it. You see your partner sitting on the lounge. You smile and give them a hug and kiss taking a moment to enjoy being back in their company. Your partner kisses you back and then asks how you would like to spend the afternoon together. Please take a moment to imagine this scenario as if it were happening to you right now.

Ambiguous
Imagine you are heading home/going to visit your partner one afternoon. Your partner is usually home at this time. You've really been looking forward to seeing them and telling them about your day. When you finally arrive, you walk up to the front door and open it. You see your partner sitting on the lounge. You smile and give them a hug and kiss taking a moment to enjoy being back in their company. However, your partner appears preoccupied with something. They look you in the eyes and say "we need to talk". Please take a moment to imagine this scenario as if it were happening to you right now.

Rejection
Imagine you are heading home/going to visit your partner one afternoon. Your partner is usually home at this time. You've really been looking forward to seeing them and telling them about your day. When you finally arrive, you walk up to the front door and open it. You see your partner sitting on the lounge. You smile and give them a hug and kiss taking a moment to enjoy being back in their company. However, your partner appears preoccupied with something. They tell you that they need to spend some time by themselves this afternoon and ask you to leave them be until they're ready. Please take a moment to imagine this scenario as if it were happening to you right now.

Vignette 8 – Romantic

For the following scenario, please imagine your partner/girlfriend or boyfriend. If you do not currently have a partner, please imagine the person from your last romantic relationship:

Control
Imagine it's been a slow week at work and you have been looking forward to the weekend. All you have wanted to do is spend some quality time with your partner. You were hoping they would be free on the weekend to spend some time with you and do something fun. You research some new ideas for what you could do together on the internet. When the weekend finally arrives, you eagerly wait for your partner to wake up so you can suggest your idea for spending time together. Later in the morning, they are still not awake, so you decide to wake them up. They reply by yawning. They agree to your idea saying, "ok let me just get ready". Please take a moment to imagine this scenario as if it were happening to you right now.

Ambiguous
Imagine it's been a slow week at work and you have been looking forward to the weekend. All you have wanted to do is spend some quality time with your partner. You were hoping they would be free on the weekend to spend some time with you and do something fun. You research some new ideas for what you could do together on the internet. When the weekend finally arrives, you eagerly wait for your partner to wake up so you can suggest your idea for spending time together. Later in the morning, they are still not awake, so you decide to wake them up. When you go to wake them up, you find out that they have already left to go somewhere else. Please take a moment to imagine this scenario as if it were happening to you right now.

Rejection
Imagine it's been a slow week at work and you have been looking forward to the weekend. All you have wanted to do is spend some quality time with your partner. You were hoping they would be free on the weekend to spend some time with you and do something fun. You research some new ideas for what you could do together on the internet. When the weekend finally arrives, you eagerly wait for your partner to wake up so you can suggest your idea for spending time together. Later in the morning, they are still not awake, so you decide to wake them up. When you explain your idea, they tell you they'd prefer to spend this weekend with their friend. Please take a moment to imagine this scenario as if it were happening to you right now.

Vignette 9 – Romantic

For the following scenario, please imagine your partner/girlfriend or boyfriend. If you do not currently have a partner, please imagine the person from your last romantic relationship:

Control
Imagine you are spending some time with your partner watching television on the lounge. Your partner is currently doing something on their phone. You start thinking about how much you love your partner and how much you enjoy their company. You'd love nothing more right now than a little bit of intimacy whether that be a cuddle, a kiss or something more. You start caressing your partner's arm and you get a little bit closer to them to get their attention. Your partner notices what you are doing, puts their phone down and looks you in the eyes smiling. They move in even closer. Please take a moment to imagine this scenario as if it were happening to you right now.

Ambiguous
Imagine you are spending some time with your partner watching television on the lounge. Your partner is currently doing something on their phone. You start thinking about how much you love your partner and how much you enjoy their company. You'd love nothing more right now than a little bit of intimacy whether that be a cuddle, a kiss or something more. You start caressing your partner's arm and you get a little bit closer to them to get their attention. However, they still appear to be on their phone and do not respond to what you are doing. Please take a moment to imagine this scenario as if it were happening to you right now.

Rejection
Imagine you are spending some time with your partner watching television on the lounge. Your partner is currently doing something on their phone. You start thinking about how much you love your partner and how much you enjoy their company. You'd love nothing more right now than a little bit of intimacy whether that be a cuddle, a kiss or something more. You start caressing your partner's arm and you get a little bit closer to them to get their attention. Your partner notices what you are doing, but they're still immersed in their phone. They tell you "I don't feel like it right now". Please take a moment to imagine this scenario as if it were happening to you right now.
