# Supplementary material for: Assessing the relationship between early maladaptive schemas and interpersonal problems using interpersonal scenarios depicting rejection
Source: PLoS One. 2023 Oct 24;18(10):e0288543. doi: 10.1371/journal.pone.0288543 (PMC10597527; doi:10.1371/journal.pone.0288543)

Appendix B: Example interpersonal vignette and response layout

For the following scenario, please imagine your partner/girlfriend or boyfriend. If you do not currently have a partner, please imagine the person from your last romantic relationship:

Imagine you are spending some time with your partner watching television on the lounge. Your partner is currently doing something on their phone. You start thinking about how much you love your partner and how much you enjoy their company. You'd love nothing more right now than a little bit of intimacy whether that be a cuddle, a kiss or something more. You start caressing your partner's arm and you get a little bit closer to them to get their attention. Your partner notices what you are doing, but they're still immersed in their phone. They tell you "I don't feel like it right now".

Please take a moment to imagine this scenario as if it were happening to you right now. Think about how you would likely interpret this situation? How are you feeling? What will you do? How do you react?

Scenario Reminder: Imagine you are spending some time with your partner watching television on the lounge. Your partner is currently doing something on their phone. You start thinking about how much you love your partner and how much you enjoy their company. You'd love nothing more right now than a little bit of intimacy whether that be a cuddle, a kiss or something more. You start caressing your partner's arm and you get a little bit closer to them to get their attention. Your partner notices what you are doing, but they're still immersed in their phone. They tell you "I don't feel like it right now". Please take a moment to imagine this scenario as if it were happening to you right now.

Think about how you would likely interpret this situation? How are you feeling? What will you do? How do you react?

Please rate the likelihood for each of the following explanations for the person's behaviour.

	Extremely unlikely	Somewhat unlikely	Neither likely nor unlikely	Somewhat likely	Extremely likely
	0 10	20 30	40 50 60	70 80	90 100
My partner's behaviour was due to something negative about them (e.g., the type of person they are, problems with their personality)					
My partner's behaviour was due to something negative about me (e.g., the type of person I am, problems with my personality)					
My partner's behaviour is unlikely to change (e.g. they always act this way towards me)					
My partner's behaviour is something that negatively affects other areas of our relationship					
My partner behaved that way on purpose rather than accidentally					

How angry or upset would you feel in this situation?

Not upset at all	Somewhat upset or angry	Mildly upset or angry	Moderately upset or angry	Extremely upset or angry

0 10 20 30 40 50 60 70 80 90 100

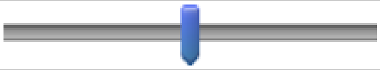

Think about how you would respond to this situation. What would you do? Please give a response based on what you would be most likely to do NOT what you think might be the best response.

____

Why would you respond this way?

____

How rejected would you feel in this situation?

Not at all	Mildly rejected	Severely rejected

0 10 20 30 40 50 60 70 80 90 100

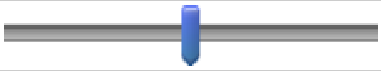

Supplement: S2 Appendix — Example layout for presentation of interpersonal vignettes and responses to vignettes including perceived rejection, cognitions, emotional distress and qualitative behavioural responses. (PDF) [file pone.0288543.s002.pdf]
