# Supplementary material for: Assessing the relationship between early maladaptive schemas and interpersonal problems using interpersonal scenarios depicting rejection
Source: PLoS One. 2023 Oct 24;18(10):e0288543. doi: 10.1371/journal.pone.0288543 (PMC10597527; doi:10.1371/journal.pone.0288543)
Supplement: Attachment — Submitted filename: Response to Reviewers.docx [file pone.0288543.s007.docx]

28-Mar-23

**Regarding: PONE-D-22-32865R1**
Assessing the relationship between early maladaptive schemas and interpersonal problems using interpersonal scenarios depicting rejection

Dear Professor Grynberg,

Thank you for the opportunity to revise our manuscript and thank you to reviewer two for their input and feedback. Please find our replies to the academic editor and reviewer in the comments below.

Sincerely,

Thomas Janovsky

School of Psychology

University of New England

Armidale NSW 2351

Australia

**Response to Academic Editor**

Journal Requirements:

Please review your reference list to ensure that it is complete and correct. If you have cited papers that have been retracted, please include the rationale for doing so in the manuscript text, or remove these references and replace them with relevant current references. Any changes to the reference list should be mentioned in the rebuttal letter that accompanies your revised manuscript. If you need to cite a retracted article, indicate the article’s retracted status in the References list and also include a citation and full reference for the retraction notice.

**REPLY: The reference list has been reviewed to ensure it is correct (see track changes). New references cited have now been added to the reference list.**

**Response to Reviewers**

**Reviewer #2:**

I appreciate the effort put in the by the authors for succinctly addressing my previous comments. After reading the revised manuscript, I just have some minor comments, which I have doubt that the authors will be able to clarify. I may have overlooked this during the first review, but in your methods, you used mixed methods design. What was the rationale behind this and how does the qualitative methods address your hypothesis?

**REPLY: The study hypotheses related to assessing the cognitive, emotional and behavioural responses to interpersonal scenarios depicting acceptance, ambiguous rejection and rejection. When measuring cognitions and emotions, questions were identical across all scenarios to ensure that variations in questions between scenarios did not impact the validity of the results. In these questions, participants were able to rate the likelihood of cognitions and distress resulting in quantitative data we could analyse statistically to test the study hypotheses.**

**However, behavioural responses to interpersonal scenarios were more challenging to assess quantitatively. Firstly, unlike cognitive and emotional responses, behavioural responses by nature depended on the external content of the scenarios which changed across control, ambiguous and rejection conditions. For example, a generic behavioural response such as “avoid them” would be confusing for some control scenarios (e.g. you text your friend to suggest an idea for catching up. They text you back suggesting a date and time. What would you do in response to this scenario?). Secondly, Weiner’s (1985) attribution theory which provided the basis for measuring cognitive responses is a well-established theory in psychology as is the use of subjective units of distress rating scales in cognitive behaviour therapy and research (e.g. Davidson et al., 2004; Woods et al., 2002). However, there are many different theories related to interpersonal behavioural – none of which were clearly superior to others for structuring questions that could be measured quantitatively for the purpose of the experiment (e.g. Interpersonal circumplex, schema coping styles). For example, Young et al.’s (2003) theory of coping styles are defined as intra-psychic responses (not necessarily behavioural responses) to a person’s early maladaptive schemas (Arntz et al., 2021; Young et al., 2003). Behavioural response constructs in many theories such as over-compensation (Schema coping styles: Young et al., 2003) and submissiveness (Interpersonal circumplex: Locke & Adamic, 2012) are also defined by whether the person reacts in a way disproportionate to the interpersonal context which in our experiment changed across conditions. Thirdly, researchers have argued that what is considered a maladaptive behavioural response varies based on context as behaviours that are maladaptive in some contexts can be adaptive in others (Fischer et al., 2021; Sih et al., 2004). For example, Fischer et al (2021) found that accepting and not acting to try to change a situation was only adaptive when the situation was unchangeable.

Given the above variability in behavioural responses due to different theories of interpersonal behaviour and changing interpersonal context and ambiguity about what behaviours can be considered “unhelpful” in changing contexts, we used an embedded mixed methods design where open ended qualitative behavioural responses were measured within a predominantly quantitative study. The qualitative behavioural responses were then converted into quantitative variables for analysis and assessment of the hypotheses investigating whether (1) participants report changing behavioural responses as the degree of rejection increases across interpersonal scenarios and (2) whether EMSs moderated the relationship between interpersonal rejection and behavioural responses.**

**To help clarify this design and rationale, the following paragraph was expanded and revised in the Experiment Stimuli section of the Methods:**

**“Emotional distress associated with each scenario was measured by using a subjective units of distress scale asking participants to rate the degree to which they would be angry or upset following the scenario on a sliding scale from 0 (Not upset at all) to 100 (Extremely upset or angry). However, unlike attributions and subjective measures of distress, behavioural responses in grounded theory are often defined by how disproportionate they are to the individual context (e.g. the schema coping style of overcompensation) which meant questions related to specific behaviours based on theory would have needed to vary across scenarios making it difficult to differentiate whether changing responses related to the changes in condition or changes in behavioural response questions. Due to the possible variability of behavioural responses across contexts, behavioural responses were measured qualitatively embedded within a predominantly quantitative design. Behavioural responses were measured by asking participants to record a written response summarising how they would likely respond to the interpersonal situation and why. The behavioural responses were then coded using inductive content analysis to create a series of binary variables scoring endorsement or non-endorsement of each behavioural response so that behaviours could be assessed by statistical analysis with respect to the study hypotheses.”**

**References**

**Arntz, A., Rijkeboer, M., Chan, E., Fassbinder, E., Karaosmanoglu, A., Lee, C. W., & Panzeri, M. (2021). Towards a reformulated theory underlying Schema Therapy: Position paper of an international workgroup. *Cognitive Therapy and Research, 45*(6), 1007–1020. https://doi.org/10.1007/s10608-021-10209-5**

**Davidson, J.R.T., Foa, E.B., Huppert, J.D., Keefe, F.J., Franklin, M.E., Compton, J.S., Zhao, N., Connor, K.M., Lynch, T.R., & Gadde, K.M. (2004). Fluoxetine, comprehensive cognitive behavioral therapy, and placebo in generalized social phobia. *Archives of General Psychiatry, 61*(10)*,* 1005-1013. https://doi.org/10.1001/archpsyc.61.10.1005.**

**Fischer, R., Scheunemann, J., & Moritz, S. (2021). Coping strategies and subjective well-being: Context matters. *Journal of Happiness Studies, 22,* 3413-3434.**

**https://doi.org/10.1007/s10902-021-00372-7**

**Locke, K. D., & Adamic, E. J. (2012). Interpersonal circumplex vector length and interpersonal decision making. *Personality and Individual Differences, 53*(6), 764–769. https://doi.org/10.1016/j.paid.2012.06.001**

**Sih, A., Bell, A.M., Johnson, J.C., & Ziemba, R.E. (2004). Behavioral syndromes: An integrative overview. *The Quarterly Review of Biology, 79*(3), 241-277. http://doi.org/10.1086/422893.**

**Weiner, B. (1985). An attributional theory of achievement motivation and emotion. Psychological Review, 92(4), 548–573. https://doi.org/10.1037/0033-295X.92.4.548**

**Woods, C.M., Chambless, D.L., & Skeketee, G. (2002). Homework compliance and behaviour therapy outcome for panic with agoraphobia and obsessive compulsive disorder. *Cognitive Behaviour Therapy, 31*(2), 88-95. https://doi.org/10.1080/16506070252959526**
