# Supplementary material for: Assessing the relationship between early maladaptive schemas and interpersonal problems using interpersonal scenarios depicting rejection
Source: PLoS One. 2023 Oct 24;18(10):e0288543. doi: 10.1371/journal.pone.0288543 (PMC10597527; doi:10.1371/journal.pone.0288543)
Supplement: Attachment — Submitted filename: Response to Reviewers.docx [file pone.0288543.s008.docx]

26-Jun-23

**Regarding: PONE-D-22-32865R1**
Assessing the relationship between early maladaptive schemas and interpersonal problems using interpersonal scenarios depicting rejection

Dear Professor Grynberg,

Thank you for the opportunity to revise our manuscript and for your input and feedback. Please find our replies in the comments below.

Sincerely,

Thomas Janovsky

School of Psychology

University of New England

Armidale NSW 2351

Australia

**Response to Academic Editor**

**Journal Requirements:**

Please review your reference list to ensure that it is complete and correct. If you have cited papers that have been retracted, please include the rationale for doing so in the manuscript text, or remove these references and replace them with relevant current references. Any changes to the reference list should be mentioned in the rebuttal letter that accompanies your revised manuscript. If you need to cite a retracted article, indicate the article’s retracted status in the References list and also include a citation and full reference for the retraction notice.

**REPLY: The reference list has been reviewed to ensure it is correct (see track changes). New references cited have now been added to the reference list.**

**Reviewer #3:**

1. I would add a sentence in the background section of your abstract to present the objective, rather than mentioning it in the method section

**REPLY: The study aims have now been mentioned in the background section rather than the methods section in the abstract:

“Background: Early maladaptive schemas (EMSs) have been theorised to contribute to reoccurring interpersonal problems. This study developed a novel experimental paradigm that aimed to assess if EMSs moderate the impact of interpersonal situations on interpersonal responses by manipulating the degree of rejection in a series of interpersonal vignettes depicting acceptance, ambiguous rejection and rejection. Method: In a sample of 158 first-year psychology students (27.2% male; 72.2% female; 0.6% other) participant responses to interpersonal scenarios were measured including degree of perceived rejection, emotional distress, conviction in varying cognitive appraisals consistent with attribution theory and behavioural responses to scenarios. Qualitative data was analysed using inductive content analysis and statistical analyses were conducted using multi-level mixed effect linear and logistic regression models using the software Jamovi (Version 1.6, Jamovi Project, 2021). Results: People reporting higher EMSs reported increased emotional distress (F(1, 156) = 24.85, p < .001), perceptions of rejection (F(1, 156) = 34.33, p < .001), self-blame (F(1, 156) = 53.25, p < .001), other-blame (F(1, 156) = 13.16, p < .001) and more intentional (F(1, 156) = 9.24, p = .003), stable (F(1, 156) = 25.22, p < .001) and global (F(1, 156) = 19.55, p < .001) attributions but no differences in reported behavioural responses. The results also supported that EMSs moderate the relationship between interpersonal rejection and perceptions of rejection (F(2, 1252) = 18.43, p < .001), emotional distress (F(2, 1252) = 12.64, p < .001) and self-blame (F(2, 1252) = 14.00, p < .001). Conclusion: Together these findings suggest that people with EMSs experience increased distress and select negative cognitions in situations where there are higher levels of rejection but that distress and negative cognitions are generally higher in people with EMSs irrespective of the situation.”**

2. Would it be possible to better argue the importance to differentiate ambiguous vs clear rejection conditions? Did you expect increased distress in these type of situations and if yes, why?

**REPLY: The following information has been added to the introduction section to help clarify the importance of differentiating ambiguous versus clear rejection and what might be expected/why based on previous study findings:

“The importance given to interpersonal contexts is argued by interpersonal theorists who have found that interpersonal behaviour is open to change across different contexts such as increased interpersonal pressures or responses from others (Locke & Adamic, 2012). For example, Farc et al. (2008) found that people who experienced physical abuse during childhood were more likely to perceive aggression in children when the cues in the environment were ambiguous. Similar findings have been found in other studies showing increased endorsement of hostility schemas to be associated with increased perceptions of hostile intentions in others when information about another person’s behaviour was ambiguous (Kim et al., 2021). Furthermore, people with higher rejection expectations reported increased withdrawal from a group when rejection in an interpersonal scenario was unambiguous compared to ambiguous (Nesdale et al., 2014; Zimmer-Gembeck & Nesdale, 2012).Together these findings suggest that individuals with EMSs are more likely to report negative responses (e.g. negative cognitions and behavioural responses) to interpersonal contexts depicting rejection and that responses are likely to become increasingly negative as the ambiguity about rejection in the scenario decreases. These findings are consistent with theoretical models depicting schemas (e.g. Schema therapy model, the rejection sensitivity model) arguing that people with stronger negative beliefs or schemas of themselves and others are more likely to perceive rejection in the environment and that perceptions of rejection are associated with higher levels of distress and more negative interpersonal behaviour responses (Young et al., 2003; Zimmer-Gembeck & Nesdale, 2012).”**

**The following sentence was also added to the Aims and Hypotheses section of the paper to re-iterate the above information with respect to the current study design:

“Measuring degree of rejection based on acceptance, ambiguous and unambiguous rejection conditions is consistent with previous studies investigating cognitive, emotional and behavioural responses in people with negative relationship expectancies such as rejection sensitivity and hostility schema (e.g. Kim et al., 2021; Zimmer-Gembeck & Nesdale, 2012).”**
